# Hepatosplenic sarcoidosis: A case report and literature review

**DOI:** 10.1097/MD.0000000000050008

**Published:** 2026-08-21

**Authors:** Weilong Wang, Yongping Luo

**Affiliations:** a Department of General Surgery I, The First Affiliated Hospital of Guangdong Pharmaceutical University, Guangzhou, Guangdong, China.

**Keywords:** diagnosis, differential, granuloma, liver, positron emission tomography-computed tomography, sarcoidosis, spleen

## Abstract

**Rationale:**

Sarcoidosis is a multisystem granulomatous disease that primarily affects the lungs. Isolated or concurrent hepatosplenic involvement, although not uncommon pathologically, is a rare clinical presentation that poses a significant diagnostic challenge and often mimics malignancy on imaging.

**Patient concerns:**

A 55-year-old asymptomatic female was admitted after the incidental discovery of pulmonary nodules during a health checkup.

**Diagnoses:**

Preoperative imaging, including contrast-enhanced computed tomography (CT) and fluorodeoxyglucose positron emission tomography/CT, suggested a high likelihood of splenic malignancy. A definitive diagnosis of hepatosplenic sarcoidosis was established after histopathological examination of both the resected spleen and a concurrent liver biopsy, which revealed noncaseating epithelioid cell granulomas.

**Interventions:**

The patient underwent laparoscopic splenectomy and intraoperative liver biopsy.

**Outcomes:**

The patient’s postoperative course was uneventful. Given the absence of symptoms and organ dysfunction, the patient was discharged without specific pharmacotherapy and was regularly monitored. To date, follow-up results have been unremarkable.

**Lessons:**

This case underscores that hepatosplenic sarcoidosis can mimic malignancy on advanced imaging studies, such as fluorodeoxyglucose positron emission tomography/CT. Therefore, sarcoidosis should be considered in the differential diagnosis of patients with multifocal splenic lesions and concurrent pulmonary nodules. We hypothesize that earlier incorporation of image-guided lesion biopsy into the diagnostic algorithm may improve diagnostic accuracy and avoid more aggressive surgical procedures in patients with multisystem nodular lesions.

## 1. Introduction

Sarcoidosis is a systemic disorder of unknown etiology characterized by the formation of noncaseating granulomas. Although over 90% of cases affect the lungs and intrathoracic lymph nodes, nearly any organ can be affected. Hepatic involvement is found in 50 to 79% of biopsies, and splenic involvement occurs in 6.7 to 77% of patients, although most remain clinically silent.^[1,2]^ Concurrent symptomatic involvement of both the liver and spleen is rare, reported in 5 to 16% of patients with extrapulmonary disease.^[3]^ Nonspecific imaging features, which often include multifocal hypodense lesions, frequently result in a misdiagnosis of lymphoma or metastatic disease, particularly when fluorodeoxyglucose (FDG) positron emission tomography (PET)/computed tomography (CT) shows high glycolytic activity. We present a case where preoperative workup strongly suggested splenic malignancy, and the correct diagnosis of hepatosplenic sarcoidosis was confirmed only after splenectomy.

## 2. Case presentation

Written informed consent was obtained from the patient for the publication of this case report in accordance with the journal’s patient consent policy. Ethical approval was obtained from the Hospital Institutional Ethics Committee (Approval No. [2026] IIT [21]).

### 2.1. Case history and examinations

A previously healthy 55-year-old woman was referred in March 2024 after a routine chest CT scan revealed multiple pulmonary nodules. She was asymptomatic, had no history of malignancy, and reported a history of long-term occupational exposure to wood dust from working in a lumber mill. Physical examination was unremarkable, and serum tumor markers were within normal limits.

**Figure 1.**
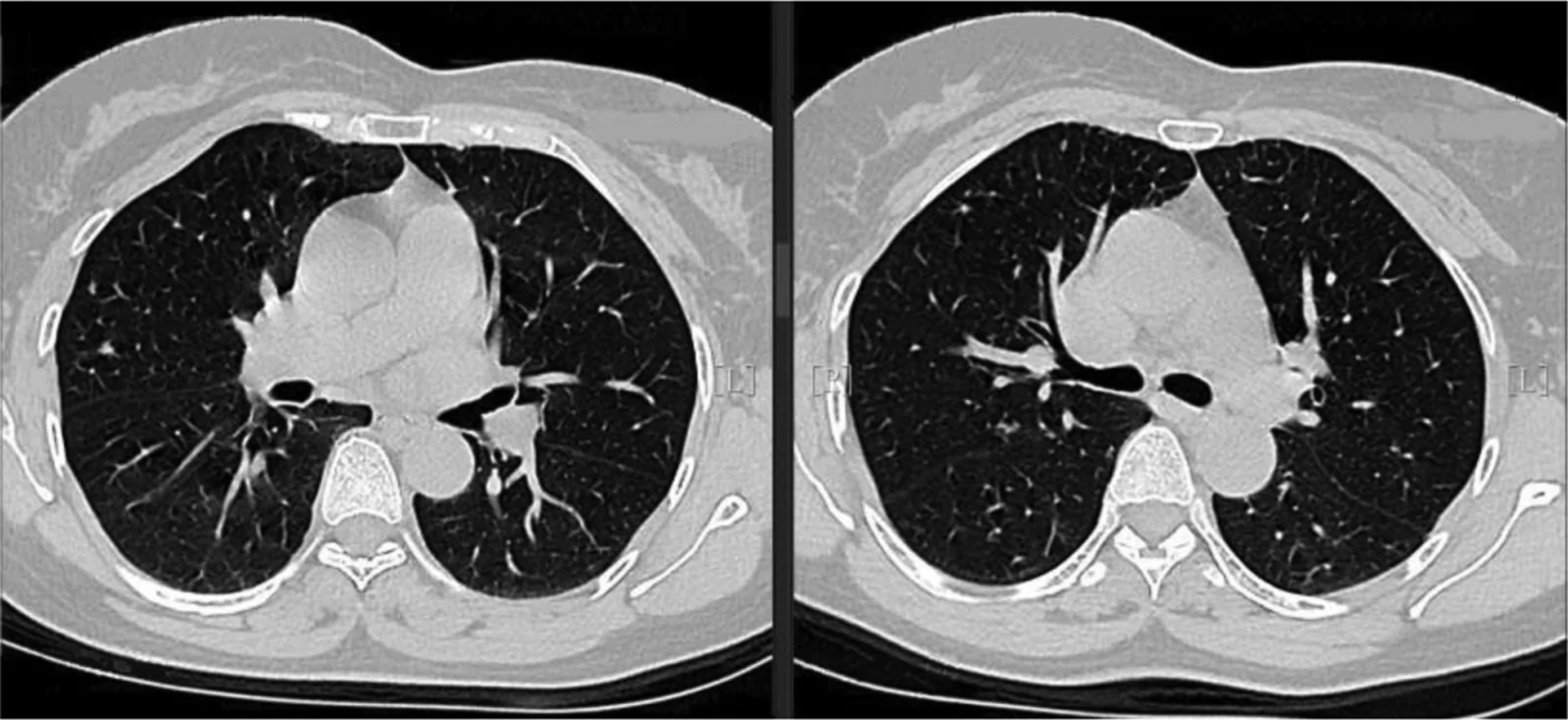
Chest CT scan shows scattered, multiple tiny solid nodules along with a few small foci of chronic inflammation and fibrotic streaks in both lungs. CT = computed tomography.

**Figure 2.**
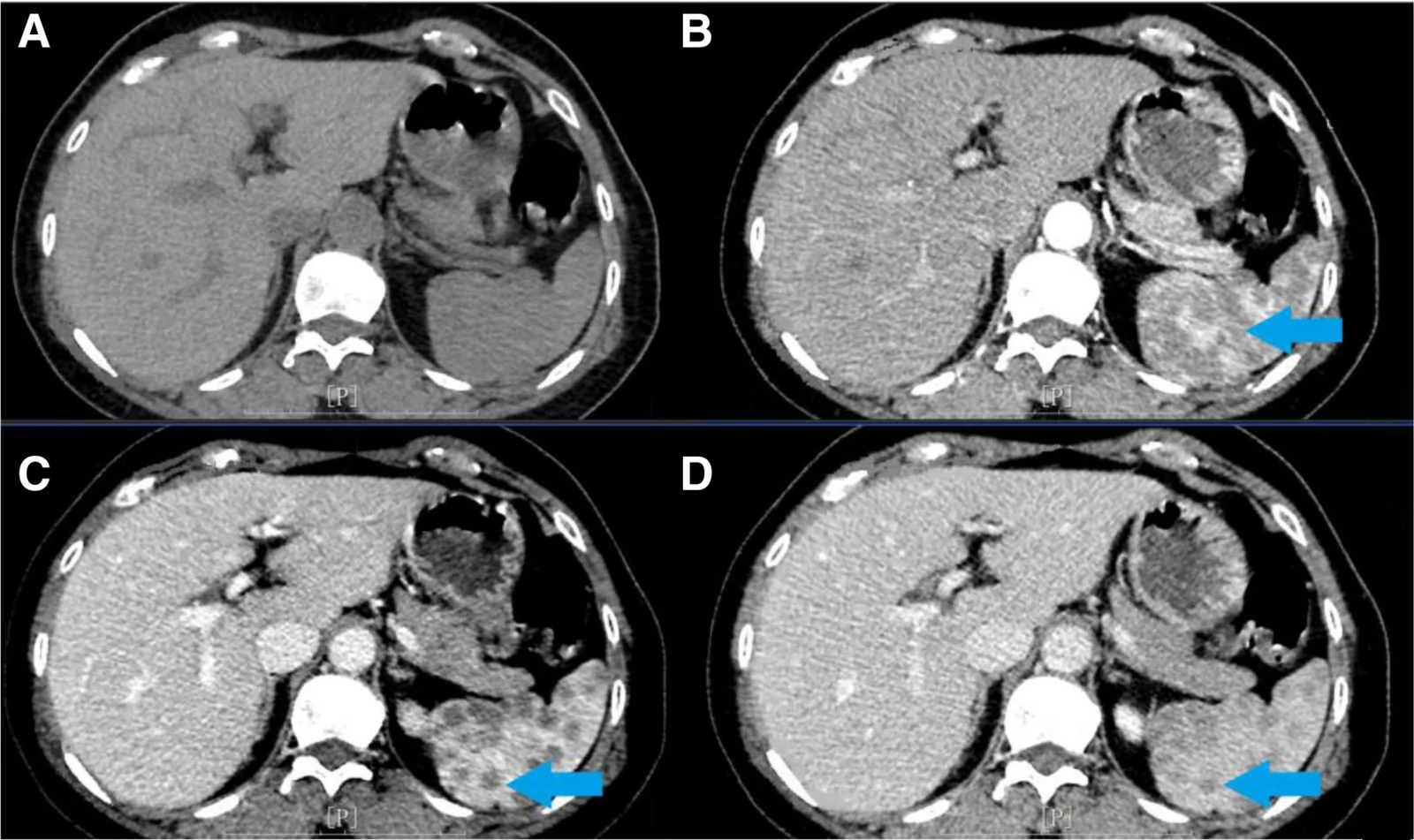
Abdominal CT scan showing a non-enlarged spleen with an irregular contour and multiple tiny hypodense nodules. (A) Unenhanced phase showing isodense nodules; (B) arterial phase showing hypodense nodules; (C) portal venous phase showing relative hypodensity; (D) equilibrium phase showing hypodense nodules. CT = computed tomography.

Contrast-enhanced CT of the chest and abdomen revealed an irregular solid nodule (approximately 12 × 10 mm) in the apical segment of the right upper lobe, along with scattered tiny solid nodules in both lungs (Fig. 1). Notably, the spleen contained multiple, diffusely distributed hypodense nodules on the portal venous and delayed phases, which were highly suggestive of a malignant process (Fig. 2).

**Figure 3.**
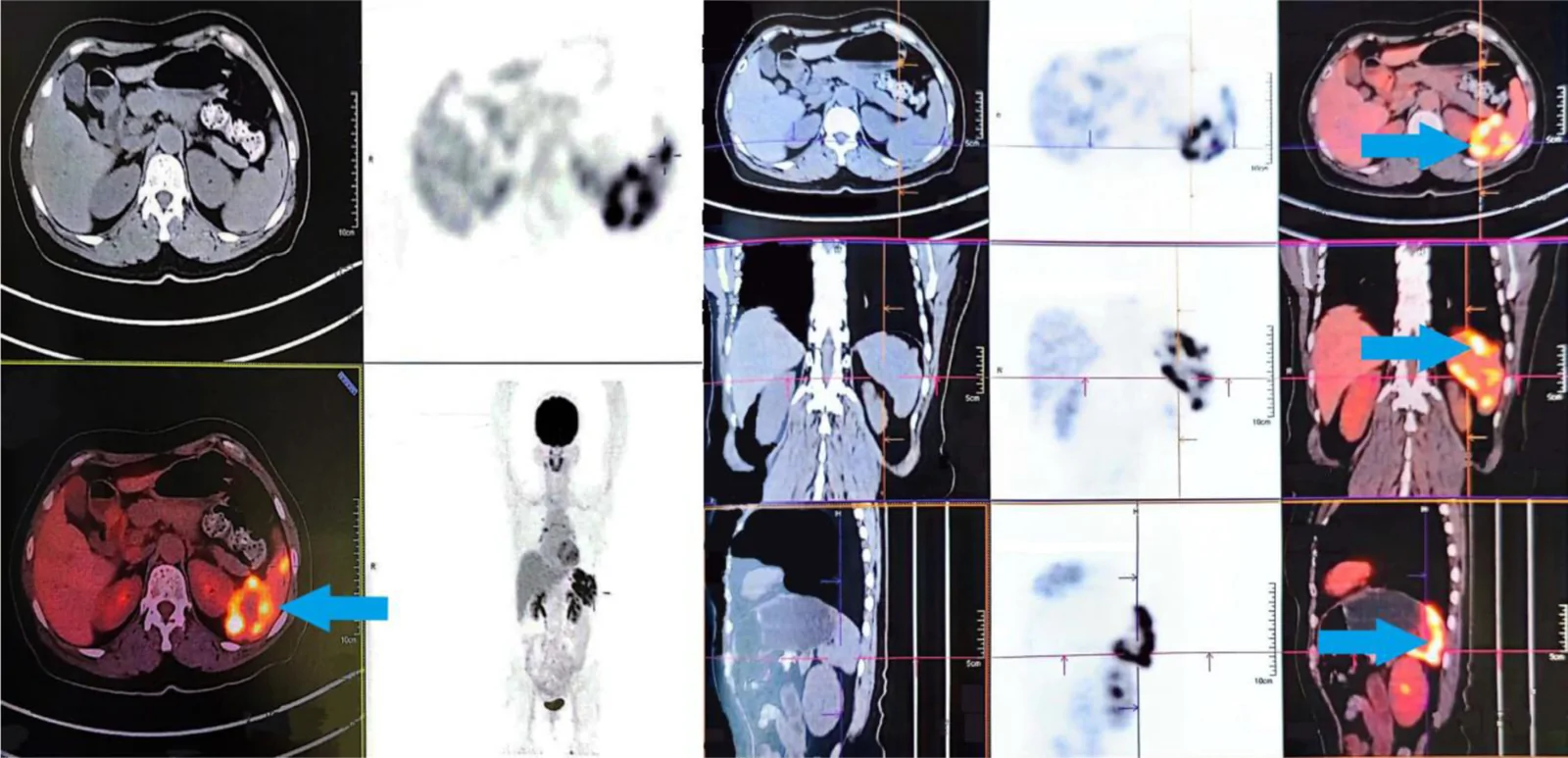
PET-CT demonstrating multiple hypermetabolic lesions in the spleen. PET-CT = positron emission tomography-computed tomography.

A subsequent whole-body FDG-PET/CT scan was obtained (Fig. 3), revealing intense FDG uptake within multiple splenic lesions, highly suggestive of malignancy (e.g., lymphoma vs metastasis). The dominant right upper lobe nodule demonstrated only mild FDG uptake, suggesting an inflammatory etiology. The liver showed no significant pathological FDG uptake.

### 2.2. Treatment

**Figure 4.**
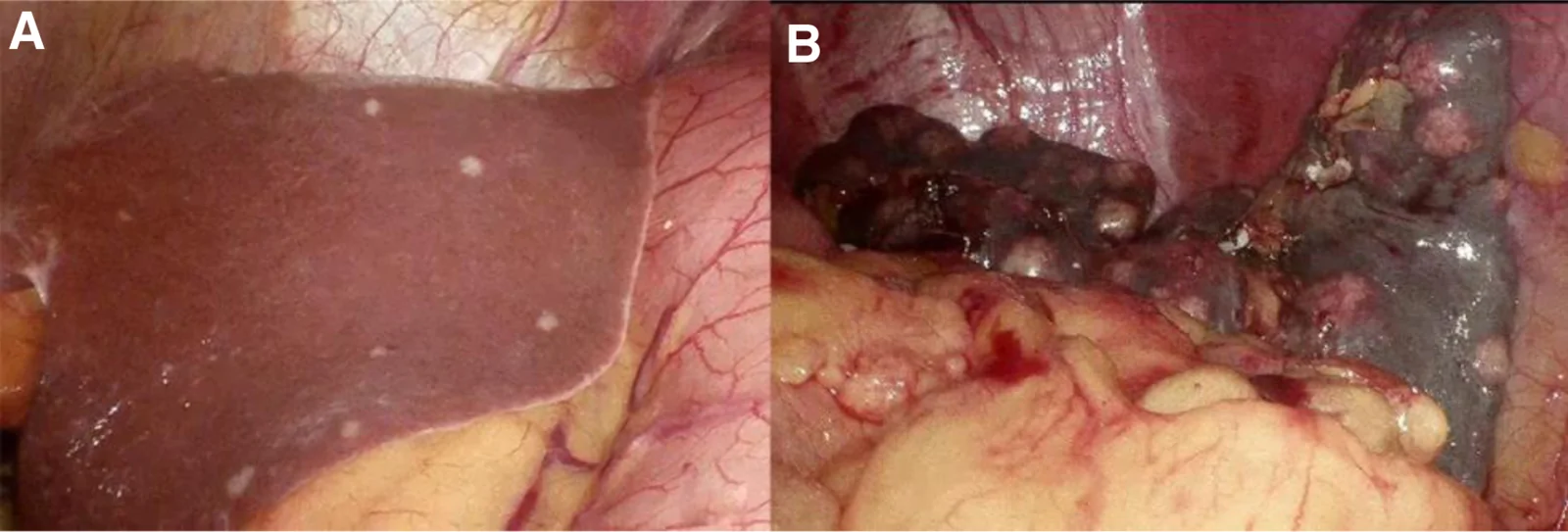
Intraoperative findings. (A) Multiple tiny nodules on the liver surface; (B) multiple nodules on the spleen surface.

Given the high suspicion for splenic malignancy, the patient underwent laparoscopic exploration. During surgery, the liver surface showed numerous millimeter-sized (1–3 mm), grayish-white nodules (Fig. 4A). The spleen appeared normal in size but had a nodular surface with adhesions (Fig. 4B). A splenectomy and a wedge biopsy of the liver were performed. The resected spleen measured approximately 13 × 5 × 3 cm, and its cut surface was multinodular.

### 2.3. Diagnosis and follow-up

**Figure 5.**
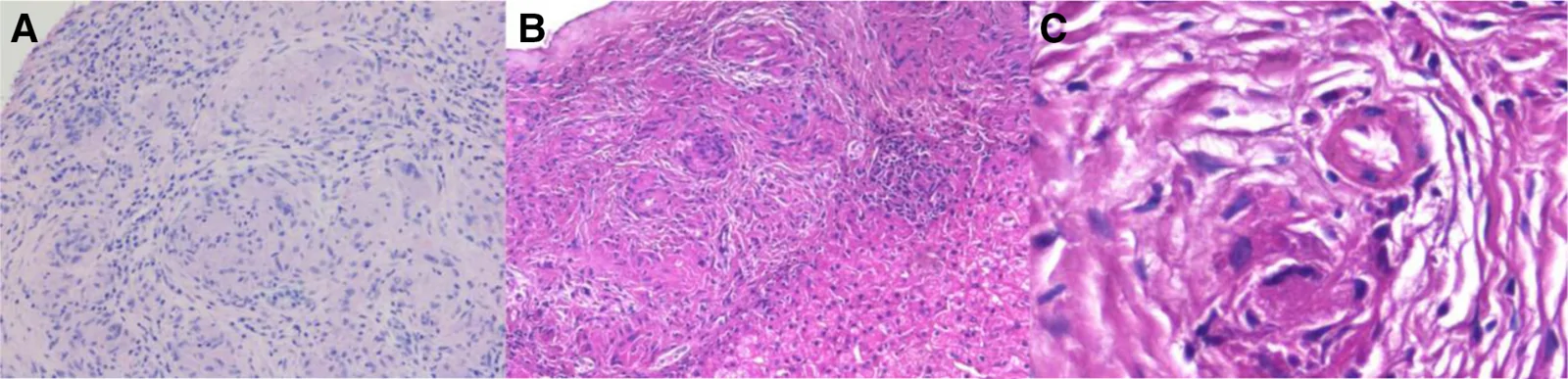
Pathological findings of the liver lesion showing noncaseating epithelioid granulomas composed of epithelioid cells and multinucleated giant cells (H&E staining). (A) Frozen section at × 40 magnification; (B) paraffin section at × 40 magnification; (C) paraffin section at × 200 magnification). H&E = hematoxylin and eosin.

**Figure 6.**
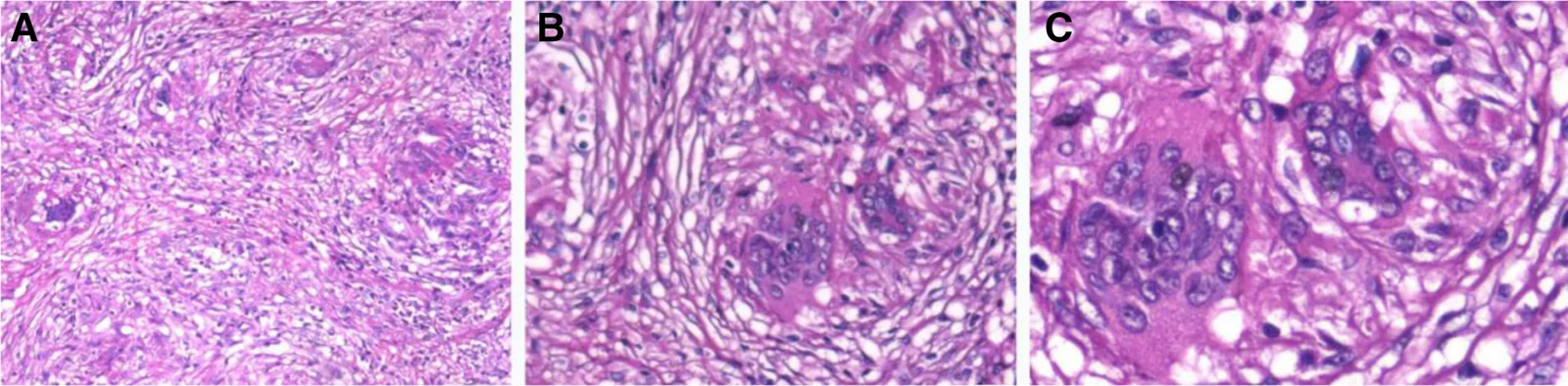
Histopathology of the splenic sections shows multiple noncaseating epithelioid granulomas composed of epithelioid cells and multinucleated giant macrophages. Special stains: acid-fast (−), Gomori methenamine silver (−), PAS (−), AB (−). (A) × 40; (B) × 100; (C) × 200). AB = Alcian blue stain, PAS = Periodic Acid–Schiff stain.

Histopathological examination of both the spleen and liver biopsy specimens revealed identical findings (Figs. 5 and 6), including well-formed, noncaseating (non-necrotizing) granulomas composed of epithelioid histiocytes and multinucleated giant cells. Special stains for acid-fast bacilli and fungi were negative. These findings were diagnostic of sarcoidosis with hepatosplenic involvement and were consistent with established diagnostic criteria.^[4]^

The patient’s postoperative recovered was uneventful. Given the absence of symptomatic organ involvement or biochemical abnormalities, the patient was managed with active surveillance without corticosteroid therapy, as spontaneous resolution occurs in a significant proportion of cases.^[5]^ The patient remains asymptomatic at follow-up visits.

## 3. Discussion

This case highlights the diagnostic dilemma posed by isolated hepatosplenic sarcoidosis. The initial clinical and radiological presentation was highly suggestive of metastatic cancer or primary splenic lymphoma, which ultimately led to a therapeutic splenectomy. The definitive diagnosis was established after histopathological examination.

Splenic involvement in sarcoidosis is most often detected incidentally on imaging. Symptomatic presentation with constitutional symptoms and marked splenomegaly occurs in up to 6% of cases.^[6]^ CT reveals splenic enlargement or nodules in up to 15% of abdominal studies, typically as single or multiple hypodense lesions larger than 1 cm, with irregular shapes and a tendency to confluence.^[7]^ According to the World Association of Sarcoidosis and Other Granulomatous Disorders organ assessment instrument,^[8]^ splenic manifestations considered suggestive of sarcoidosis include: highly probable, based on biopsy findings compatible with sarcoidosis; and probable, indicated by low-attenuation splenic nodules on CT, hyperattenuating nodules on PET/scintigraphy, or splenomegaly on examination or imaging.^[9]^

Published case reports on concurrent liver and spleen involvement in sarcoidosis showed that affected patients could have various clinical presentations that frequently mimic lymphoma or metastatic cancer, often requiring biopsy or surgical resection for definitive diagnosis. Yonenaga et al reported a 24-year-old woman with a history of right ovarian cancer found to have multiple FDG-avid nodules in the spleen and liver. She was initially diagnosed with metastatic ovarian cancer and underwent laparoscopic splenectomy and partial hepatectomy. The final histopathological examination revealed noncaseating epithelioid cell granulomas consistent with sarcoidosis.^[10]^ Makis et al described a 60-year-old woman with no significant past medical history who presented with hematuria. An FDG-PET/CT scan demonstrated extensive FDG-avid lymphadenopathy along with innumerable nodules in the liver, spleen, and lungs, suspicious for disseminated malignancy.^[11]^ Finally, a biopsy revealed non-necrotizing well-formed granulomas supporting the diagnosis of sarcoidosis. Uslu et al presented a 34-year-old man with widespread cutaneous and subcutaneous FDG-avid lesions on PET/CT scan, which mimicked metastatic malignancy or lymphoma. The histological analysis of the axillary lymph node biopsy specimen confirmed noncaseating granulomas, leading to the final diagnosis of multisystemic sarcoidosis.^[12]^

Some patients with sarcoidosis might present with atypical systemic symptoms, asymptomatic hepatic and splenic nodules, hypercalcemia, massive splenomegaly, pancytopenia, or rarely, severe complications such as portal hypertension and Budd–Chiari syndrome caused by granulomatous involvement of hepatic veins. Fiuza et al reported a 66-year-old woman with chronic hepatosplenomegaly and persistent high cholestatic enzymes, but no lymphadenopathy. Extensive workup excluded infectious, autoimmune, and hematologic causes. A liver biopsy revealed non-necrotizing granulomas consistent with sarcoidosis.^[13]^ Fakili et al presented a 68-year-old woman with abdominal pain and dyspnea. Laboratory tests showed hypercalcemia. Abdominal images demonstrated diffuse multiple nodular hyperintense lesions in the liver and spleen. The liver biopsy confirmed hepatosplenic sarcoidosis.^[14]^ Saito et al reported a 22-year-old woman with chronic abdominal distention for 3 years. Further examinations showed severe pancytopenia and a massively enlarged spleen (13 × 24 cm). A liver biopsy demonstrated noncaseating epithelioid granulomas, confirming the diagnosis of sarcoidosis.^[15]^

Minami et al described a 71-year-old woman with cutaneous sarcoidosis who presented with a liver mass and Budd–Chiari syndrome. The liver mass was found to be hepatocellular carcinoma.^[16]^ Van Brusselen et al presented a 7-year-old boy who had severe Budd–Chiari syndrome and received liver transplantation. Seven years later, the patient developed recurrent venous outflow obstruction in the allograft. Histopathological examination revealed diffuse noncaseating granulomas that led to the diagnosis of sarcoidosis.^[17]^ Fauter et al reported 12 cases with illnesses related to liver injury, such as ascites, esophageal varices, and hepatic encephalopathy. Most of them required a liver biopsy to diagnose sarcoidosis.^[18]^

Very rarely, liver and spleen sarcoidosis was diagnosed during the workup for pulmonary nodules. Zhang et al reported a 53-year-old woman with a previous history of left lung adenocarcinoma who had undergone surgery. Nine months postoperatively, she was found to have multiple lesions in the right upper lung, bilateral pleura, spleen, and liver. Histopathological study confirmed the diagnosis of sarcoidosis.^[19]^

Overall, all these case reports and our present case report show that patients with sarcoidosis can present with distinct and atypical clinical symptoms. Affected patients might have multiple nodular lesions simultaneously involving several organ systems, which could easily lead clinicians to suspect metastatic malignancy based on conventional imaging and PET/CT scans. A biopsy and histopathological examination are commonly required to find noncaseating granulomas, which are the diagnostic cornerstone for sarcoidosis. Recent reports also highlight the value of minimally invasive diagnostic techniques, such as endoscopic ultrasound-guided fine-needle aspiration, in avoiding unnecessary surgical approaches, such as splenectomy.^[20]^ Liver and spleen involvement in sarcoidosis can carry a risk of significant complications, such as portal hypertension, cirrhosis, variceal bleeding, and rarely, liver failure.^[18,21,22]^ A liver biopsy might be considered in clinically stable patients for accurate diagnosis and appropriate management to avoid permanent damage to important organ systems.

In this case, the multiple low-attenuation splenic nodules on CT and increased FDG uptake on PET/CT were key features consistent with the World Association of Sarcoidosis and Other Granulomatous Disorders criteria for probable splenic involvement. FDG-PET/CT, while invaluable in oncology, is known to have a significant pitfall in the context of sarcoidosis. Activated inflammatory cells within sarcoid granulomas exhibit high avidity for FDG, leading to false-positive results that mimic malignancy.^[23]^ In this case, PET/CT failed to identify the liver lesions later seen intraoperatively, underscoring its limitation in detecting micronodular disease, a finding supported by other reports in which hepatic involvement is often an incidental discovery.^[24]^

The diagnosis of sarcoidosis rests on 3 pillars: a compatible clinical and radiological presentation, histological evidence of noncaseating granulomas, and the exclusion of other granulomatous diseases (e.g., tuberculosis, fungal infections).^[25]^ Our patient fulfilled all 3 criteria, with the diagnosis further supported by negative special stains.

This case also prompts reflection on the management strategy. The intraoperative discovery of characteristic liver surface findings and a frozen-section result consistent with noncaseating granulomas could have prompted earlier consideration of sarcoidosis. We propose that a more conservative approach might be attempted in managing similar cases, such as avoiding splenectomy but seeking a definitive diagnosis through nodular biopsy and histopathological examination first, after a thorough discussion with the patient and the patient’s family members.

The nature of the patient’s pulmonary nodules remains indeterminate without a lung biopsy. While they may represent pulmonary sarcoidosis, the possibility of unrelated inflammatory nodules cannot be ruled out. Her occupational exposure to wood dust is a notable environmental factor, consistent with theories on sarcoidosis pathogenesis involving inhaled antigens.^[1]^

Sarcoidosis management is individualized. Treatment of asymptomatic hepatic sarcoidosis is typically observational, as progression to significant liver disease is uncommon.^[24,26]^ This rationale is consistent with our decision for surveillance. Symptomatic disease or signs of organ damage warrant therapy, with corticosteroids as the first line, ursodeoxycholic acid for cholestatic features, steroid-sparing immunosuppressants as second-line, and anti-tumor necrosis factor-α biologics for refractory cases.^[27]^

Here, we presented this case to remind clinicians that sarcoidosis can involve multiple organ systems, and affected patients can present with various symptoms. The limitation of our study was its single case report. The experience learned, such as biopsy versus surgical splenectomy, might not be applicable to other patients. Patients with multiple nodular lesions involving several organ systems should be carefully evaluated, weighing the risks and benefits of different diagnostic and management approaches.

## 4. Conclusions

Hepatosplenic sarcoidosis is a notorious mimicker of malignancy. In patients with multifocal splenic lesions, particularly when presenting concurrently with pulmonary involvement or other systemic features suggestive of granulomatous disease, sarcoidosis should be included in the differential diagnosis. A nodular biopsy (percutaneous, when feasible and safe) for histopathological examination might be considered as the initial diagnostic workup. We propose that clinicians maintain a high index of clinical suspicion and employ a multidisciplinary approach to synthesize clinical, radiological, and histopathological findings in patients with evidence of multisystem involvement.

## Author contributions

**Conceptualization:** Weilong Wang, Yongping Luo.

**Data curation:** Weilong Wang.

**Formal analysis:** Weilong Wang, Yongping Luo.

**Funding acquisition:** Weilong Wang.

**Investigation:** Weilong Wang.

**Methodology:** Weilong Wang.

**Project administration:** Yongping Luo.

**Resources:** Weilong Wang, Yongping Luo.

**Validation:** Weilong Wang, Yongping Luo.

**Visualization:** Weilong Wang.

**Writing – original draft:** Weilong Wang.

**Writing – review & editing:** Yongping Luo.
